# Feasibility, Acceptability, and Preliminary Efficacy of an App-Based Meditation Intervention to Decrease Firefighter Psychological Distress and Burnout: A One-Group Pilot Study

**DOI:** 10.2196/34951

**Published:** 2022-06-08

**Authors:** Thaddeus W W Pace, Katharine H Zeiders, Stephanie H Cook, Evelyn D Sarsar, Lindsay T Hoyt, Nicholas L Mirin, Erica P Wood, Raquel Tatar, Richard J Davidson

**Affiliations:** 1 Division of Biobehavioral Health Science College of Nursing University of Arizona Tucson, AZ United States; 2 Department of Psychiatry College of Medicine University of Arizona Tucson, AZ United States; 3 Department of Psychology College of Science University of Arizona Tucson, AZ United States; 4 Norton School of Family and Consumer Sciences College of Agriculture and Life Sciences University of Arizona Tucson, AZ United States; 5 Department of Social and Behavioral Sciences School of Global Public Health New York University New York, NY United States; 6 Department of Biostatistics School of Global Public Health New York University New York, NY United States; 7 Department of Psychology Fordham University Bronx, NY United States; 8 Healthy Minds Innovation Madison, WI United States; 9 Department of Psychology University of Wisconsin Madison, WI United States

**Keywords:** firefighter, meditation, smartphone app, anxiety, cortisol, digital health, mobile health, mHealth, mental health, burnout, stress management

## Abstract

**Background:**

Firefighters are often exposed to occupational stressors that can result in psychological distress (ie, anxiety and depression) and burnout. These occupational stressors have only intensified with the onset of the COVID-19 pandemic and will likely persist in the postpandemic world.

**Objective:**

To address occupational stressors confronting firefighters, we pilot tested a novel, cost-effective, smartphone app–based meditation intervention created by Healthy Minds Innovations that focused on mindfulness (awareness) training along with practices designed to cultivate positive relationships (connection), insight into the nature of the self (insight), and a sense of purpose in the context of challenge (purpose) with a sample of professional firefighters from a large metropolitan area in southwestern United States.

**Methods:**

A total of 35 participants were recruited from a closed online group listserv and completed the self-guided 10-unit meditation app over the course of 10 days, at 1 unit per day. We assessed anxiety symptoms, depression symptoms, burnout, and negative affect as well as saliva diurnal cortisol rhythm, an objective indicator of stress-related biology, before and after use of the meditation app.

**Results:**

This study demonstrated the meditation app was both feasible and acceptable for use by the majority of firefighters. We also found significant reductions in firefighters’ anxiety (*P*=.01), burnout (*P*=.05), and negative affect (*P*=.04), as well as changes in cortisol diurnal rhythm, such as waking cortisol (*P*=.02), from before to after use of the meditation app.

**Conclusions:**

Our study findings call for future research to demonstrate the efficacy of this meditation app to reduce psychological distress and burnout in firefighters.

## Introduction

Along with other first responders, firefighters are often exposed to occupational stressors such as interpersonal conflict and workplace fairness issues [1], as well as resuscitations and other clinical emergencies that can be psychologically traumatic. Exposure to these occupational stressors increases the risk that firefighters will develop stress-related chronic illnesses, such as anxiety and depression, posttraumatic stress disorder (PTSD), and chronic pain [2]. Throughout the COVID-19 pandemic, occupational stressors that drive the risk for stress-related illnesses in firefighters have only intensified [3,4] and will likely continue in the postpandemic world. To address psychological distress (ie, anxiety and depression) resulting from exposure to occupational stressors confronting firefighters, we pilot tested a novel, cost-effective, smartphone app–based meditation intervention that focused on mindfulness, connection with others, and compassion for the self and others.

The term “meditation” refers to contemplative practices that bring mental capacities and processes under greater voluntary control not just during but also between practice sessions [5,6]. Mindfulness is a popular style of meditation that is designed to cultivate the quality of nonjudgmental awareness in the present moment [6,7]. The benefits of structured mindfulness meditation interventions to reduce anxiety and depression, as well as to improve various stress-related biomarkers, have been demonstrated in several different populations, ranging from adolescents to cancer survivors to police officers [8-10]. A few published studies suggest that mindfulness meditation may also improve various aspects of health for firefighters [11-14], and 2 studies, both with the same intervention, tested an intervention delivered to firefighters virtually (ie, not in person) [13,15]. There has also been descriptive research on dispositional mindfulness in firefighters that supports the use of mindfulness meditation in this group. Specifically, dispositional mindfulness (or awareness) has been inversely associated with firefighters’ PTSD symptoms, features of anxiety and depression, perceived stress, and suicide risk [7,14,16,17]. Together, these findings suggest that interventions intended to cultivate mindfulness and awareness in firefighters, including by way of meditation, may be effective to reduce their anxiety and depression.

Besides mindfulness, available evidence suggests that other styles of meditation may be worthwhile for firefighters. One group of structured meditation interventions collectively called “compassion meditation” (eg, Cognitively-Based Compassion Training) are designed to promote compassion for the self and others. Previous research has demonstrated that compassion meditation interventions improve anxiety, depression, and perceived stress in different populations [18-20]. Although we are not aware of any studies to date that have explored the benefits of compassion meditation for firefighters, perceived social connection (ie, social support) has been found to buffer against the effects of trauma exposure and PTSD symptoms in firefighters [21]. This suggests that firefighters may benefit from a meditation intervention that actively cultivates the perception of the importance of connection to others in addition to nonjudgmental awareness in the present moment (ie, mindfulness). We therefore tested a meditation app intervention that included both mindfulness and compassion. Our selection of content for the app was guided by Lazarus and Folkman’s theory of stress and coping [22], which has been used previously to study health in firefighters [23], as well as research indicating the importance of mindfulness and perceived social connection for firefighter health [7,14,16,17,21].

We decided to study an asynchronous app-based approach instead of an in-person meditation intervention because conditions during the COVID-19 pandemic (when the study was conducted) prevented large gatherings of people. We also selected an app-based approach to reduce intervention costs. While in-person meditation interventions with trained interventionists can be relatively costly (ie, approximately US $60 per person per group session), asynchronous app-based interventions have fixed development costs that can be recouped over time.

Guided by the stress and coping theory as well as prior research on how stress affects the health of firefighters [1,7,14,16,17], we assessed self-reported anxiety symptoms, depression symptoms, burnout, and negative affect before and after the 10-day meditation intervention. We also included an objective biological outcome of stress, diurnal cortisol rhythm, which is the profile of the cortisol concentration in saliva over the course of the day that is normally high in the morning, peaks 30-60 minutes after wakening (cortisol awakening response [CAR]), and is low at night, indicating healthy hypothalamic-pituitary adrenal (HPA) axis function [24]. Relative disruptions in diurnal cortisol rhythm (eg, high bedtime levels, a flatter diurnal slope from waking to bedtime, high overall concentration across the day) have been associated with self-reported stress and other negative psychological health outcomes (eg, depression) [24,25]. Meditation interventions have been found in prior research to improve or stabilize diurnal cortisol rhythm [26,27], especially in those at risk after exposure to stress [28]. Besides predicting that the meditation app would be feasible to implement with firefighters, we hypothesized that symptoms of anxiety, depression, burnout, and negative affect would decline from before to after use of the meditation app. We also hypothesized that changes in diurnal cortisol rhythm, suggestive of improved HPA axis function, would be evident after use of the meditation app.

## Methods

### Participants

Participants were career firefighters from a large metropolitan city in southwestern United States (N=35) who were recruited via flyers emailed to a firefighters’ union closed listserv. To be eligible for the study, participants had to (1) be actively working as a firefighter in the geographic region of interest; (2) work a typical 24 hours on/48 hours off schedule; (3) have a smartphone with requisite knowledge to operate their device, including for internet access; (4) have no vacation time scheduled for the duration of the study; (5) report no current symptoms of COVID-19; and (6) not be taking any corticosteroid medication during the study period. Interested firefighters completed an online survey to verify their eligibility and provided informed consent before enrollment and after being informed about the length and purpose of the study in real time by the study staff.

### Ethics Approval

All procedures for this research were approved by the University of Arizona Human Subjects Protection Program (Project #2005659631).

### Design and Procedures

This study used a 1-group pretest-posttest design. After giving consent online, participants were contacted by study personnel via telephone to discuss the study protocol and the study start date. Participants were then sent a study packet via mail that included an information sheet describing the daily activities, saliva sampling protocol, instructions on downloading the meditation app, and a “spit kit” to collect saliva samples, which included 6 vials (with labels to record sample collection time), straws, and prepaid postage to send samples back to the laboratory.

The study protocol was conducted over the course of 16 days, including preassessments, intervention, and postassessments. To aid with compliance to the study protocol, study personnel texted or called participants each day of the study to remind them of the daily tasks and answer any concerns or questions. Participants completed aspects of the study, including data collection, from their workplace or at home from on smartphones. On day 1, participants completed a closed online baseline survey via Qualtrics software that assessed demographic characteristics and psychosocial and behavioral constructs. On days 2 and 3, participants completed a brief daily online survey in the mornings that included self-report assessments (eg, anxiety and depression), COVID-19-related stress, and sleep quality (findings regarding the latter two are reported elsewhere). They also provided saliva samples upon waking, 30 minutes after waking, and at bedtime on both days (6 total samples). On days 4 to 13 (10 days), participants completed the 10-minute meditation segments guided by the app and a brief online daily survey (both administered via their smartphones). On days 14 and 15, participants ended use of the meditation app but continued with the online self-report data collection and completed 2 more additional days of saliva sampling (upon waking, 30 minutes after waking, and at bedtime, with a total of 6 samples). On day 16, participants completed a final online survey. Around this time, participants were also contacted by study staff to gather comments about the meditation app and the study procedures. Participants were then instructed to ship their samples to the research team using the overnight return envelopes provided by the study team. Participants were compensated US $200 if they completed over 60% of the daily online surveys and US $160 if they completed less than 60%. Study information was stored on password-protected servers that were only accessible by the research team.

### Intervention

**Figure 1:**
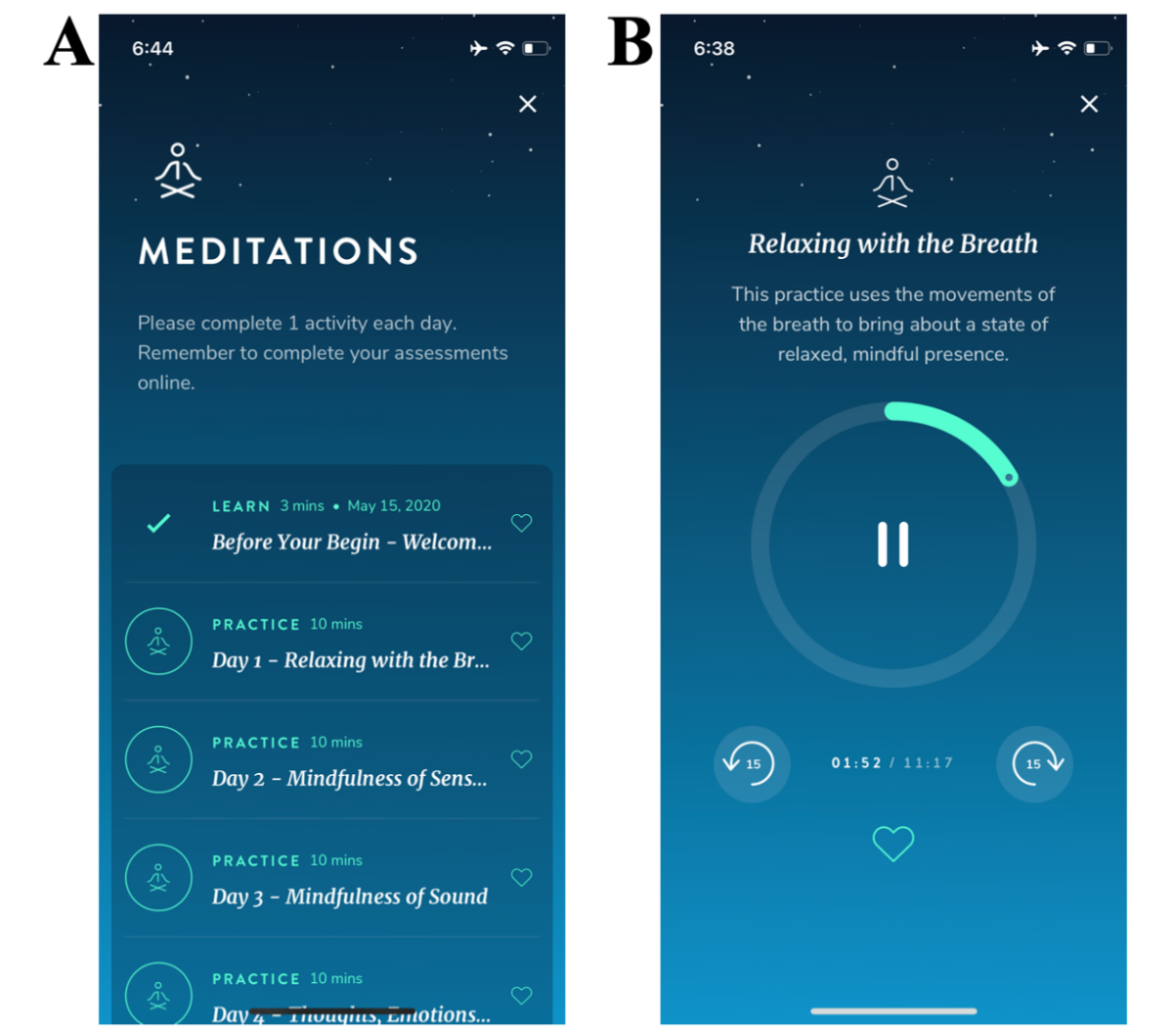
The Healthy Minds Innovations app home screen (A) and a session screen (B).

The smartphone app–based meditation intervention we tested was created by Healthy Minds Innovations (HMI) [29]. The app consists of 10 individual 10-minutes sessions, with 1 session per day conducted over 10 consecutive days. Our selection of content in the version of the app for this study with firefighters was guided by Lazarus and Folkman’s theory of stress and coping [22], which has been used previously to guide a firefighter study [23], as well as research indicating the importance of mindfulness and perceived social connection for firefighter health [7,14,16,17,21]. Accordingly, the app provided audio recordings on the constructs of awareness (or mindfulness) and connection (with others/compassion). The app also included sessions on insight (including compassion for the self and others) and purpose (eg, finding meaning in challenges experienced by the self and others), which collectively promoted lessons of connection. The app remained frozen and unchanged during the study period. Screenshots of the app homes screen and an app session are shown in Figure 1.

After enrolling and completing the initial assessment, study staff provided a link and unique access code to each participant to download the meditation app created by HMI to their phone (either iPhone or Android). After accessing the meditation app, participants were presented with a welcome screen, followed by a screen that presented them with the option to turn on a notification allowing the app to remind them to practice at a particular time of day, if desired. Participants then landed on a “meditations” page, from which they were able to select the day 1 module. Including the first day, the app contained 10 individual modules that covered the overarching constructs of awareness, connection, insight, and purpose. A description of each module by day is presented in Table 1. Participants were able to select a module only after they completed the prior module. Participants were instructed to complete 1 module per day over the course of the 10-day intervention period but were allowed to catch up on units if they missed a day.

**Table 1:** The Healthy Minds Innovation app with construct focus and description by day.

Day	Topic title	Construct focus	Description
1	Relaxing with the breath	Awareness	Attending to the breath while staying in the present moment with the body
2	Mindfulness of sensations	Awareness	Attending to sensations while in the present moment with the body
3	Mindfulness of sound	Awareness	Attending to sounds while staying in the present moment
4	Thoughts, emotions, and the breath	Awareness	Attending to thoughts, emotions, and breath while staying in the present moment with the body
5	Seeing the good in ourselves	Connection	Shifting awareness of the body to our natural talents and strengths
6	Gratitude	Connection	Realizing and giving thanks for our connections to others
7	Compassion in difficult situations	Connection	Realizing our innate wish to be happy and free from suffering and extending that wish to others
8	Question your assumptions	Insight	Examining our unconscious beliefs that influence how we see ourselves and how we view other people and situations around us
9	Values in difficult times	Purpose	Applying our values in the moment to help us stay grounded and resilient
10	The meaning of adversity	Purpose	Finding meaning in challenges experienced by us and others

### Quantitative Outcomes

Self-report instruments containing 46 individual items were tested extensively by 2 members of the research team (authors NLM and EPW) for usability and technical functionality in Qualtrics before being administered to the participants. Qualtrics evaluated the responses to all items for completeness before participants were able to select the “finished” button at the bottom of the questionnaires screen. Participants were also able to review their responses before clicking “finished.” Each enrolled participant was allowed to respond to the set of items only once at each assessment time point, which was managed by Qualtrics using cookies. The university logo was displayed at the top of the Qualtrics screen.

#### Anxiety Symptoms

The Patient Reported Outcomes Measurement Information System (PROMIS) Short Form v1.0– Emotional Distress–Anxiety 8a was used at baseline and at the end of the intervention to assess firefighters’ experiences of emotions such as fear, stress, and anxiety within the past 7 days. This scale was developed with the Item Response Theory [30]. Responses were rated on a 5-point Likert scale ranging from 1 (never) to 5 (always), and a total raw score was obtained by summing the scores of all items. Summative raw scores can range from 8 to 40 and are converted to *t* scores to compare to population norms, with higher scores indicating greater anxiety symptoms. This scale demonstrated adequate reliability at the baseline (α=.91) and end (α=.88) assessments.

#### Depressive Symptoms

PROMIS Short Form v1.0–Emotional Distress–Depression– Short Form 8a was used at baseline and at the end of the intervention to assess the frequency of firefighters’ experiences of emotions such as worthlessness, hopelessness, and sadness. This scale was also developed with the Item Response Theory [30], and responses were rated on a 5-point Likert scale ranging from 1 (never) to 5 (always). The total raw score is obtained by summing the scores of all items ranging from 8 to 40 and converting them to *t* scores to compare to population norms, with higher scores indicating greater depressive symptoms. This scale demonstrated adequate reliability at the baseline (α=.92) and end (α=.87) assessments.

#### Burnout

The short version of the 10-item Burnout Measure was used to assess firefighters’ symptoms of burnout at baseline and at the end of the intervention [31]. The scale asks participants to rate their feelings about their work (eg, tired, disappointed with people, hopeless), and responses are rated on a 7-point Likert scale ranging from 1 (never) to 7 (always). A score of 0 to 2.4 indicates a very low level of burnout, a score between 2.5 and 3.4 indicates danger signs of burnout, and a score between 3.5 and 4.4 indicates burnout. The scale has been used with firefighters in prior work [32], and in this study, demonstrated adequate reliability at the baseline (α=.86) and end (α=.84) assessments.

#### Negative Affect

The Positive and Negative Affect Schedule (PANAS-SF) [33] was used assess firefighters’ negative affect on at baseline and at the end of the intervention. Participants were asked to respond to 10 items (eg, distressed, upset) using a 5-point Likert scale ranging from 1 (very slightly or not at all) to 5 (extremely). Scores range from 10 to 50, with higher scores reflecting higher levels of affect in either domain. For negative affect, the internal consistency was shown to be adequately reliable for day 2 (α=.84) and day 14 (α=.82).

#### Salivary Cortisol

Saliva was gathered for 2 consecutive days before the intervention (days 2 and 3) and after the intervention (days 14 and 15); on each sampling day, participants provided samples upon waking, 30 minutes after waking, and at bedtime. Participants passively drooled through a straw into a 2 mL polypropylene tube and labeled each tube with the time and date. Participants were instructed not to eat, drink, or brush their teeth 30 minutes before each sample and to keep completed samples in the refrigerator throughout the study period. Participants shipped the saliva samples to the lab, which were then stored at −20 C. Saliva samples were batch assayed in duplicate according to manufacturer instructions for cortisol concentrations using an enzyme immunoassay kit (Salimetrics). Inter- and intra-assay coefficients of variability were 8.16% and 7.74%, respectively. In line with prior work [34], the following cortisol parameters were assessed: waking level (sample 1), bedtime level (sample 3), CAR (the difference between sample 2 and sample 1), diurnal slope (the difference between sample 3 and sample 1), and area under the curve (AUC) to assess total cortisol output across the day. AUC was calculated using the trapezoidal method.

Given that diurnal cortisol patterns are impacted by sleep quality and night awakening [35], firefighters were scheduled to begin the study so that their first day of cortisol sampling occurred on their second day off from work, and their second day of sampling occurred on their first day on work. This eliminated firefighters having to take a waking sample following a shift (for which a waking sample would be hard to determine given that most firefighters are woken up multiple times in the night due to emergency calls).

### Determination of Feasibility and Statistical Analytic Plan

Intervention feasibility was defined as 80% or more of the participants completing 9 or more of the 10 meditation app sessions over the 10-day intervention period. Acceptability was determined by comments about the app and study procedures derived from poststudy interviews. Feasibility to collect saliva samples and self-report data was defined as collection of 80% or more of saliva samples or self-report variables at appropriate time points, according to the study protocol. Pre- to postintervention changes in study outcomes were analyzed with pairwise comparison *t* tests for self-reported (ie, burnout, anxiety symptoms, depressive symptoms, negative affect) and cortisol measures.

## Results

### Participants

The entire study protocol was conducted between June and July 2020. Majority of the firefighters identified as men (n=30, 86%) and were 37 years old on average (SD 8.04). Firefighters identified their racial/ethnic background as White (n=22, 63%); Hispanic or Latino (n=8, 23%); Black or African American (n=1, 3%); Native American, American Indian, or Alaskan native (n=1, 3%); and multiracial/ethnic (n=1, 3%). Two of the 35 firefighters identified their racial/ethnic background as other (ie, human, American). On average, the participants had been working as firefighters for 9.8 years (SD 7.58). In terms of education, the participants reported some college, vocational, or technical school (n=6, 17%); associate’s degree (n=11, 31%), bachelor’s degree (n=13, 37%), and some advanced work or a master’s degree (n=5, 14%). Most firefighters reported their relationship status as married (n=23, 66%), while 9% (n=3) were single, 9% (n=3) divorced, and 17% (n=6) in a relationship but not married (Table 2).

**Table 2:** Participants’ demographic information (N=35).

Participant characteristics		Values
**Gender,n (%)**		
	Men	30 (86)
	Women	5 (14)
**Ethnic/racial identification, n (%)**		
	White	22 (63)
	Hispanic or Latino	8 (23)
	Black or African American	1 (3)
	Native American, American Indian, or Alaskan Native	1 (3)
	Multiracial/ethnic	1 (3)
	Other	2 (6)
**Education, n (%)**		
	Some college	(48)
	Bachelor’s degree	13 (37)
	Advanced (eg, Master's, PhD)	5 (14)
**Marital status, n (%)**		
	Married	23 (66)
	Single	3 (9)
	Divorced	3 (9)
Age (years), mean (SD)		36.7 (8.03)
Firefighting experience (years), mean (SD)		9.81 (7.57)

### Feasibility and Acceptability

Study compliance was high; 33 (94%) of the 35 participants completed the postintervention self-report assessments. As for intervention engagement, 80% (n=28) of the participants completed either 9 or all 10 of the app segments; the remaining participants completed 8 segments (n=2), 5 segments (n=1), 2 segments (n=2), and 1 segment (n = 2). Additionally, 94% (n=33) of the participants collected at least one home saliva sample at both the initial assessment and the end assessment. In poststudy interviews, multiple participants reported that they found use of the app worthwhile. No participants reported concerns with the app or other study procedures. No technical problems or privacy breaches occurred during the study.

Given the interest in preliminary intervention effects, we performed attrition analyses to determine if participants who completed 9 or 10 segments (n=28) differed from those who completed less than 9 (n=8) on relevant demographic characteristics and baseline indicators. No differences emerged for race/ethnicity, gender, relationships status, education level, or age; however, firefighters who completed 9 or 10 HMI segments had been working for more years (mean 10.92, SD 8) than those who completed fewer than 9 HMI segments (mean 5.36, SD 2.87; *t*_28.28_=2.99, *P*=.006). No differences emerged on baseline measures of burnout, anxiety and depressive symptoms, negative affect, bedtime cortisol, cortisol awakening response, diurnal slope, or cortisol AUC. There were differences on waking cortisol (*t*_31_=−2.67, *P*=.01); those who participated in most HMI segments had lower waking cortisol levels (mean 0.25, SD 0.10) than those who completed fewer HMI segments (mean 0.38, SD 0.14) at the preintervention assessment.

For the baseline and end in-home saliva sampling protocols, most participants aligned with the schedule (n_baseline_=22; n_end_=19). There were, however, firefighters whose schedules did not align with that of the study protocol. All firefighters’ schedules were on a 24-hour work/48-hour off rotation; however, it is common for firefighters to pick up additional overtime shifts, so they may only have 1 day off in between. If firefighters worked both cortisol sampling days (n_baseline_=2) or if they misreported their work schedules (n_baseline_=2), their cortisol data were not used. If firefighters worked the day prior to the first day of saliva sampling and worked during the second day of saliva sampling, only their second day of samples were used (n_baseline_=5, n_end_=2). Similarly, if firefighters worked the day prior to their first day of sampling but were off on the second day of sampling, only their second day of samples were used (n_end_=2). If firefighters were off the day prior to the first day of saliva sampling but worked on both saliva sampling days, only the first day’s samples were used (n_baseline_=1, n_end_=1). Table 3 shows the final number of participants for each of the cortisol outcomes.

**Table 3:** Pairwise comparison *t* tests from pre- to postintervention.

Outcomes			Sample size, n		Preintervention, mean (SD)		Postintervention, mean (SD)		Pairwise *t* test (*P* value)		Effect size (*d*)
**Self-reported outcomes**											
	Burnout	33		2.48 (0.79)		2.29 (0.69)		*t*_32_=2.03 (*P*=.05)		0.35	
	Anxiety symptoms	33		53.00 (7.99)		49.65 (7.27)		*t*_32_=2.70 (*P=.*01)		0.47	
	Depressive symptoms	33		48.40 (7.65)		47.23 (7.23)		*t*_32_=1.35 (*P=*.19)		0.24	
	Negative affect	26		13.88 (4.48)		12.65 (3.74)		*t*_25_=2.17 (*P*=.04)		0.43	
**Physiological outcomes**											
	Waking cortisol (µg/dL)	19		0.288 (0.120)		0.221 (0.084)		*t*_18_=2.61 (*P*=.02)		0.60	
	Bedtime cortisol (µg/dL)	19		0.049 (0.053)		0.042 (0.060)		*t*_18_=0.34 (*P*=.74)		0.08	
	CAR^a^	17		0.091 (0.143)		0.082 (0.101)		*t*_16_=0.32 (*P*=.75)		0.08	
	Cortisol diurnal slope	16		−0.016 (0.010)		−0.012 (0.007)		*t*_15_=−.1.31 (*P*=.21)		0.33	
	Cortisol AUC^b^	16		2.60 (1.21)		2.11 (0.63)		*t*_15_=2.127 (*P*=.05)		0.53	

^a^CAR: cortisol awakening response.

^b^AUC: area under the curve.

### Quantitative Outcomes

To examine pre- to postintervention change in outcomes, pairwise comparison *t* tests were performed on self-report (ie, burnout, anxiety symptoms, depressive symptoms, negative affect) and physiological measures (ie, waking cortisol, bedtime cortisol, CAR, diurnal slope, cortisol AUC; Table 3). Significant pre- to postintervention differences were found for burnout, anxiety symptoms, and negative affect (Table 3, Multimedia Appendix 1). In particular, firefighters reported lower burnout, lower anxiety symptoms, and lower negative affect postintervention. Pre- to postintervention differences were not found for depressive symptoms. Significant pre- and posttest differences were also found for measures of waking cortisol and cortisol AUC (Table 3, Multimedia Appendix 1), with firefighters exhibiting lower waking cortisol and lower cortisol AUC after the intervention. There were no pre- and postintervention differences in bedtime cortisol, CAR, or diurnal slope.

## Discussion

### Principal Results

The goal of this study was to determine the feasibility and acceptability of a novel smartphone-based meditation app for use by firefighters. In addition, we hypothesized that symptoms of anxiety, depression, burnout, and negative affect would decline after use of the app. We also hypothesized that changes in diurnal cortisol rhythm, suggestive of improved HPA axis function, would result from before to after use of the meditation app. We found that use of the app was both feasible and acceptable by a sample of career firefighters. We were also able to collect reliable psychological self-report outcomes and saliva samples from most of our sample, despite the highly variable shift work schedule. In terms of pre- and postintervention outcomes, we found a reduction in self-reported anxiety and burnout symptoms, as well as negative affect. We also observed statistically meaningful (or strong trend) reductions in several measures related to diurnal cortisol rhythm, including waking saliva concentrations of cortisol and cortisol area under the curve, over the course of the day. Together, these findings suggest that use of the HMI smartphone-based meditation app may be associated with improvement in objective stress-related physiological indicators and self-reported psychological factors. We believe these findings, although preliminary, are noteworthy because they provide evidence of the utility and accessibility of a cost-effective app-based meditation intervention to reduce distress and burnout in firefighters.

### Limitations

This study has several limitations. First, the sample was relatively small, although it was appropriately sized to investigate feasibility and acceptability, and the power was adequate for the outcomes assessed [36]. Second, the study only included a single arm, with no waitlist control or other comparison group, such as an active attention control. Future research with the meditation app for firefighters should include an appropriate active comparison group that controls for likely nonspecific intervention elements (eg, learning something new from a smartphone app over a period of 10 days) to further establish the efficacy of the meditation app for firefighters. Future studies may also work to determine whether daily text reminders to engage with the app have an impact on results. Third, participants were from the same large metropolitan area. Future studies should involve firefighters from both smaller and larger urban settings, as well as rural settings, to ensure generalizability of findings regarding meditation app efficacy in different regions. Finally, as this was an initial study, we did not control for multiple comparisons. Future studies with larger sample sizes and preregistered hypotheses can address this limitation.

### Comparison With Prior Work

Firefighters typically experience occupational stressors that include not only events related to fighting fires but also interpersonal conflict, workplace fairness issues, resuscitations, and other clinical emergencies [1]. Events involving emergency medical care are likely to be encountered more frequently by firefighters than fighting fires, and in some cases, these may be experienced together (eg, when working automobile accidents). Such events can be psychologically traumatic and, along with interpersonal stressors that generate occupational stress, can activate stress-related biological pathways. Repeated activation over time of these pathways by stressor exposure may increase the risk for stress-related chronic illnesses, including diseases that are leading causes of morbidity and mortality in firefighters (eg, cardiovascular disease) [37]. Indeed, a recent systematic review found that occupational stressors experienced by firefighters are associated with various illness states, including PTSD, hypertension, and musculoskeletal pain, as well as objective markers of stress-related biology (ie, heart rate variability) [1]. In this study, we found that use of the meditation app was associated with a decline in self-reported anxiety, burnout, and negative affect, as well as changes in diurnal cortisol rhythm (ie, lower morning cortisol and cortisol AUC), suggesting that the app may reduce the effects of occupational stressors on stress biological pathways in firefighters.

Anxiety is often comorbid with PTSD [38], and as such, any beneficial effects of the meditation app on anxiety may also have a beneficial effect on PTSD symptom severity experienced by many firefighters. We also observed an effect on firefighters’ burnout, suggesting that the meditation app may lessen the effect of occupational stressor exposures on firefighters. When firefighters take time for reflection, self-compassion, and connection, they can be more present and positive about their work. Finally, the decline in negative affect we observed was also notable because it highlights how the meditation app may exert a beneficial effect on daily emotional processes, and by extension, on chronic health conditions that may be exacerbated by negative affect [39].

While prior studies have explored the potential benefits of meditation interventions for firefighters, these studies have focused almost exclusively on mindfulness meditation [11-13,15]. Mindfulness meditation is notably different from other forms of meditation, including the HMI meditation app that we studied here that cultivates both mindfulness and feelings of social connection. Although a meditation intervention created by Joyce and colleagues [13,15] called Resilience@Work Mindfulness includes some components of compassion, these components represent only about a sixth of Resilience@Work Mindfulness and only 3 out of 18 of its guided practice audio tracks. This contrast with the HMI meditation app, which includes 5 out of 10 units that involve compassion for the self and/or others. While the HMI app does incorporate mindfulness and attention training, these skills are developed so they can be leveraged in later units to address analytic concepts of compassion for self and connection with others. Thus, the content of the HMI meditation app is distinctly different from mindfulness meditations studied before with firefighters [11-13,15]. In addition, the HMI meditation app is smartphone based, while previous efforts with firefighters have been limited to in-person meditation interventions [11,12] or tablet or computer-facilitated interventions [13,15].

Along with psychological distress, many firefighters experience physical symptoms related to chronic pain that are secondary to musculoskeletal disorders [2], sometimes due to occupational injuries. Although we did not assess pain or other somatic conditions in this study, future studies with this meditation app would do well to include measures of physical health along with distress. While there are many reasons why firefighters experience occupational injuries, the available evidence suggests that injury risk for firefighters may be predicted by psychological distress (ie, depression) [40-43]. The association between occupational injury and psychological distress in firefighters may involve effects of stress exposure on depression and anxiety [44], which in turn may impact awareness and other aspects of dispositional mindfulness [45-47] and therefore workplace safety [48-50].

### Conclusions

In this study, we demonstrated the feasibility and acceptability of a 10-day meditation app created by HMI in a sample of career firefighters. We also found that anxiety, burnout, and negative affect improved from before to after use of the meditation app, and we noted changes in various indicators of cortisol diurnal rhythm. These findings suggest that a meditation app with high potential for widespread distribution may improve firefighter health at a low cost. Additional research is needed to demonstrate the efficacy of this intervention versus an active attention control and to show how the meditation app can positively impact other aspects of health that are relevant for firefighters, including physical health and occupational injury risk.

## Appendix

Multimedia Appendix 1 Burnout (panel A), anxiety (panel B), negative affect (panel C), and 2 indicators of diurnal cortisol rhythm (waking saliva concentrations of cortisol [Panel D] and cortisol area under the curve [Panel E]) decreased from before to after use of the Healthy Minds Innovations app in firefighters. Error bars indicate standard error of the mean. **P*≤.05, ***P*≤.01.

## Abbreviations

AUC: area under the curve
CAR: cortisol awakening response
HMI: Healthy Minds Innovations
HPA: hypothalamic-pituitary adrenal
PANAS-SF: Positive and Negative Affect Schedule
PROMIS: Patient Reported Outcomes Measurement Information System
PTSD: posttraumatic stress disorder
