# Supplementary figures and images for: Feasibility, Acceptability, and Preliminary Efficacy of an App-Based Meditation Intervention to Decrease Firefighter Psychological Distress and Burnout: A One-Group Pilot Study
Source: JMIR Form Res. 2022 Jun 8;6(6):e34951. doi: 10.2196/34951 (PMC9218885; doi:10.2196/34951)

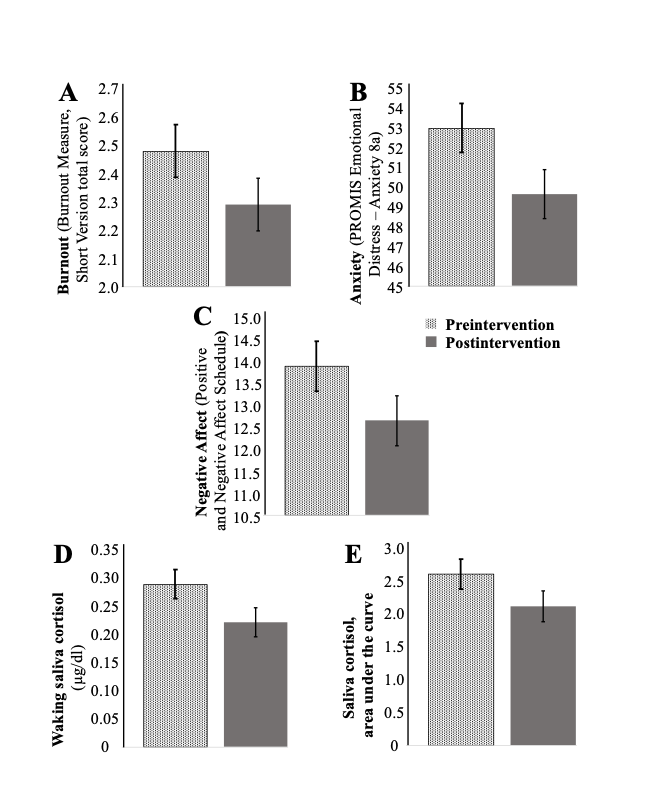

Supplement: Multimedia Appendix 1 [file formative_v6i6e34951_app1.png]
